# The use of telecytology for the evaluation of thyroid nodules fine-needle aspiration biopsy specimens: a systematic review

**DOI:** 10.1007/s40618-024-02378-3

**Published:** 2024-05-04

**Authors:** V. Oteri, S. Piane, E. Cocci

**Affiliations:** 1 Endocrine Unit, Department of Clinical and Experimental Medicine, University of Catania, Garibaldi-Nesima Hospital, Catania, Italy; 2 Department of Medicine and Surgery, University of Milano-Bicocca, Milan, Italy; 3 Department of Clinical and Experimental Medicine, Marche Polytechnic University, Ancona, Italy

**Keywords:** Telecytology, Telecytopathology, Thyroid nodules, Fine-needle aspiration biopsy, Rapid-on-site-evaluation

## Abstract

**Purpose:**

Fine needle aspiration biopsy (FNAB) is currently the gold standard for diagnosis and treatment of thyroid nodules, but the growing need for anatomic pathology services in small communities is becoming a challenge. Telecytology (TC) is defined as the electronic transmission of cytological digital images, and allows for the collection of samples, primary diagnosis, and other applications without the physical presence of a pathologist. Our aim is to systematically report, summarize, and critically analyze the most up to date applications of TC to thyroid nodules FNAB evaluation.

**Methods:**

We performed a systematic literature review by searching PubMed, Embase, and Cochrane Library databases. Only studies published in peer-reviewed scientific journals were included. Data were extracted using the PICO framework and critically analyzed. PRISMA guidelines were applied, and the risk of bias in the included studies was assessed using the ROBINS-I tools. The methodological quality was assessed following GRADE criteria.

**Results:**

We included 13 observational studies, resulting in a total of 3856 evaluated FNAB specimens. The majority of studies (63.6%) showed an excellent concordance rate of diagnosis via TC and conventional cytology. TC can be used to perform preliminary assessment of samples with a concordance rate ranging from 74 and 100%, showing a significant reduction of the non-diagnostic rate. Image quality was referred to as perfect or nearly perfect in most cases, regardless of telecytology technique.

**Conclusion:**

Telecytology could be a valuable implementation for thyroid FNAB evaluation both for primary diagnosis and preliminary assessment of samples.

**Supplementary Information:**

The online version contains supplementary material available at 10.1007/s40618-024-02378-3.

## Introduction

Thyroid nodules are a common finding among the general population [1, 2]. Their prevalence has been estimated at around 4% if assessed via palpation, which is the least sensitive diagnostic method, and autopsy data from patients with no history of thyroid diseases have shown a prevalence of 50%, especially after age 50 [3, 4]. The risk of malignancy for a nodule is between 7 and 15%, and its diagnosis should include careful clinical evaluation, laboratory testing, ultrasound imaging, and cytological exam [5, 6].

Fine needle aspiration biopsy (FNAB) currently plays a major role in the management of thyroid nodules, as it is safe, simple, and cost-effective [1, 7–9]. Indeed, there was a 16% annual growth in the number of thyroid FNAB performed in the USA between 2006 and 2011 [10]. Given that, the need for real-time anatomic pathology services has been growing, and it is becoming a challenge to provide it in small communities, as it is not cost-, resource-, or time-efficient [2, 11].

Telecytology (TC) has the potential to meet this need. It is generally described as the electronic transmission of cytological digital images, and it has proven to be useful in many aspects [2]. Among them, one of the most promising is rapid on-site evaluation (ROSE), which is a preliminary assessment method that occurs at the point of care to provide immediate diagnostic feedback during procedures, in order to ensure specimen (aspirate or biopsy) adequacy and quality [12–14].

Three distinct TC methods can be identified: static TC, which is the transmission of static images representing only selected areas of glass slides; dynamic TC, which allows for the continuous transmission of the microscope field of view in real-time as a remote control or a cytotechnologist maneuvers the microscope; and virtual microscopy or whole slide imaging (WSI), which consists of the transmission of the whole glass slides previously scanned by dedicated machinery [15–17].

Studies have shown there is high concordance between traditional cytology reports and TC, allowing for the collection of samples and primary diagnosis without the physical presence of a pathologist [14, 18–20]. Despite this data, some pathologists may still be reluctant to use virtual images to analyze cytological samples. For a reliable diagnosis, pathologists must discern cellular structures and three-dimensional features, using micro-focusing and viewing the entire slide accurately. Telecytology, even with the cutting-edge WSI, simplifies microscope images, reducing the potential fields and layers available for analysis. Furthermore, despite the highest resolution, image quality through telecytology may not be comparable to the direct observation of samples with an optical microscope. On the other hand, computer-assisted analysis of digitalized images could aid in the detection of abnormalities, potentially reducing human error [21].

During the last decade, many studies have focused on the application of telecytology to thyroid FNAB cytologic evaluation, proving its effectiveness and reliability, along with additional possible benefits of using this technique.

One of the challenges in the field of thyroid FNAB is the differential diagnosis between benign and malignant tumors, such as between benign thyroid nodules and papillary thyroid carcinoma (PTC); this distinction is based on the evaluation of particular cytological criteria, including cellular nuclear features. These characteristics are sometimes difficult to recognize, and this can lead to misdiagnosis or unnecessary surgical excision. TC can be applied efficiently to the evaluation of cytological criteria as it can visualize the same cellular features as conventional cytology [1].

Furthermore, TC has numerous possible applications, including second opinions on challenging cases, international collaboration, and discussion among experts in the field, but can also provide high-quality medical education, training, quality assurance, and proficiency testing [2, 22–24]. One example is a study based on the iPath platform, where solo pathologists practicing in remote and rural areas had the ability to receive consults from experts via TC, which was crucial for effective diagnosis [25].

Another example is the one by Stergiou et al. [22], who introduced a web-based platform for teaching cytology to their students and received very positive feedback, such as increased interest and attentiveness.

However, there are no records of a systematic review that summarizes all this evidence. We hypothesize that TC has a significant impact on clinical practice by allowing for remote diagnosis and reducing nondiagnostic biopsies, therefore increasing the collaboration between endocrinologists and pathologists.

The aim of our systematic review is to report, summarize, and critically analyze, both quantitatively and qualitatively, the most up to date applications of telecytology for thyroid FNAB cytologic evaluation.

## Materials and methods

### Literature search strategy

We conducted this review following the Preferred Reporting Items for Systematic Reviews and Meta-Analyses (PRISMA) guidelines [26] and methodological advice from the Cochrane Handbook for Systematic Reviews of Interventions [27]. Our main aims were: (1) to investigate the characteristics of telecytology techniques applied to thyroid nodules fine-needle aspiration biopsies, (2) to identify the degree of concordance between diagnosis via telecytology and conventional cytology, (3) to evaluate the validity of telecytology for preliminary assessment of the adequacy of the samples, and (4) to assess the quality of image transmission with telecytology.

A comprehensive search was performed on three medical electronic databases (PubMed, Embase, and Cochrane Library) until January 13, 2023. To achieve the maximum sensitivity of the search strategy, we combined the terms referring to the following topics: telecytology, telemedicine, cytology, thyroid, and fine-needle aspiration biopsy. We tailored searches to individual databases, using appropriate controlled vocabulary indexing and natural language search terms; the full search strategy is available in Appendix 1 of the Online resource. Additionally, for further identification of potentially relevant studies, and to ensure completeness of this review, we looked at the reference lists of the included articles, previous literature reviews on the topic, and top hits from Google Scholar. To avoid overlapping with other ongoing reviews, we first searched on the PROSPERO website for any similar reviews and then registered our review protocol (ID CRD42023424924).

### Selection criteria

Eligible studies for our systematic review included those investigating every aspect of the application of telecytology to thyroid nodules fine-needle aspiration biopsies and reporting all outcomes. Studies dealing with other conditions in addition to thyroid nodules were excluded if thyroid nodules cases were less than 25% of the total cases, and if specific results on thyroid nodules were not available separately.

**Fig. 1:**
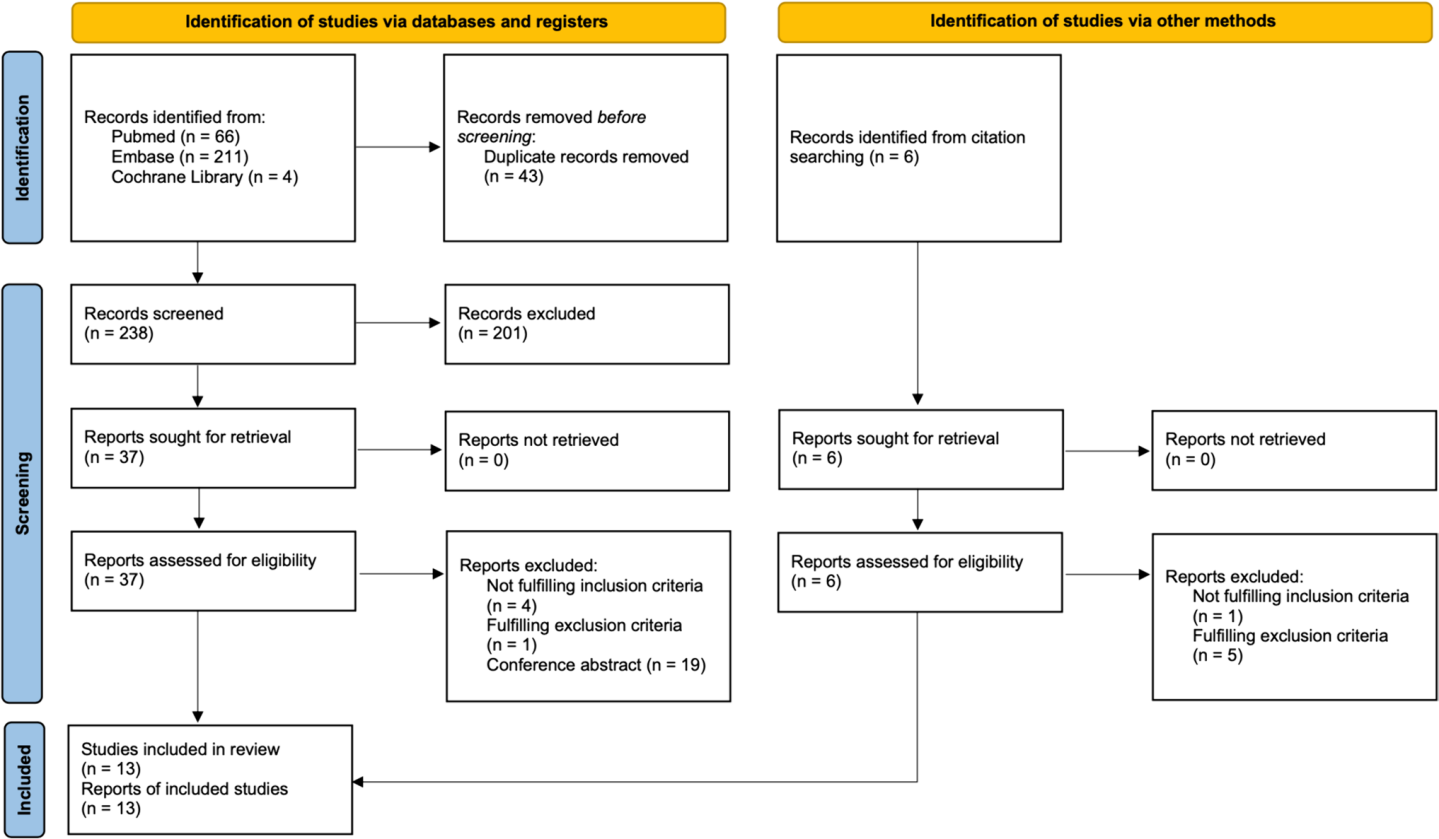
PRISMA flow diagram of study selection

Titles and abstracts underwent primary screening and were deemed eligible if they included any level of evidence, were published in peer-reviewed journals, and were written in English. Studies were excluded if the data were inaccessible, missing, not available as a full text, or not well-reported. Duplicates, abstracts, case reports, conference presentations, reviews without original data, editorials, and expert opinions were also excluded. Full-text screening was then performed on the remaining studies. At the beginning of the procedure, each investigator read the abstract or full text of all the articles, selected the relevant ones according to both inclusion and exclusion criteria, then compared the results with the other investigators, erring on the side of inclusion. After 4 weeks, the same studies were reread to establish agreement between the investigators about article selection. Two authors (V.O. and S.P.) performed the search and evaluated the articles independently. Cases of doubt were solved by consensus with a third author (E.C.). Figure 1 depicts PRISMA flow diagram of study selection.

### Data extraction and criteria appraisal

All data were extracted from the articles’ text, tables, and figures using the Population, Intervention, Comparison, Outcome (PICO) framework [28] and included at a minimum the title, authors, year of publication, country, study design, telecytology technique and setting, sample size, study population, case characteristics, intervention and comparator (where applicable), outcomes, and conclusions. One investigator extracted the data from the full-text articles and entered them into an Excel spreadsheet with structured tables to qualitatively analyze each study. The other investigator independently double-checked the extraction of primary data from all the articles. Two investigators independently reviewed each article (S.P. and E.C.). Discrepancies between the two reviewers were resolved through discussion until they reached a consensus. The final results were reviewed by the senior investigator (V.O.).

### Risk of bias assessment

Risk of bias (RoB) assessment of the full-text of the selected studies was performed according to the ROBINS-I tool [29] for non-randomized trials, which evaluates seven domains of bias to reach an overall RoB judgment (Low, Moderate, Serious, Critical)﻿.

Two authors performed the assessment (S.P. and E.C.) independently. Any discrepancy was discussed and resolved with the senior investigator (V.O.). Risk of bias assessment informed the GRADE assessment and supported the results section. Table 1S in the Online resource outlines the RoB assessment.

### Study quality assessment

The research methodology quality assessment was completed by applying the GRADE approach at the outcome level [30]. Using the ROBINS-I tool to assess RoB, evidence from observational studies begins at a grade of high-quality, as outlined by Schünemann et al. [31]; the assessment continues with subsequent evaluation of five factors that can decrease the quality of evidence and three factors that can increase it. This leads to an overall evaluation of a body of evidence as one of four grades: High: we are very confident that the true effect lies close to the estimate of the effect; Moderate: we are moderately confident in the estimated effect, the true effect is likely to be close to the estimate, but there is a possibility it is substantially different; Low: our confidence in the estimated effect is limited, the true effect may be substantially different from the estimate; Very low: we have very little confidence in the estimated effect, the true effect is likely to be substantially different from the estimate. The assessment was performed by two authors (S.P. and E.C.) independently. Any discrepancy was discussed and resolved with the senior investigator (V.O.). GRADE assessment informed the Summary of Findings table. Table 2S in the Online resource outlines the quality assessment.

## Results

### Study characteristics

We included 13 studies that evaluated the use of telecytology for thyroid FNAB assessment, accounting for a total of 3856 thyroid cases [2, 13, 14, 20, 23, 32–39]; four studies [34–36] did not focus exclusively on thyroid nodules, but also assessed samples from other organs (such as lymph nodes, lung, liver, pancreas, head and neck, and salivary glands). Ten studies were retrospective [2, 13, 23, 32–38], two were prospective [14, 39], and one consisted of two different phases: the first being retrospective, the second prospective [20]. Five studies were conducted in the USA [32–34, 37, 39], three in Greece [13, 23, 38], two in Turkey [20, 36], one in Colombia [35], one in Portugal [2], and one in Brazil [14]. All the studies were published between 2009 and 2022, with eight of them published after 2018 [13, 14, 20, 34–37, 39].

Five studies used real-time microscopy [14, 32–34, 39], four used static images [13, 23, 36, 38], three used virtual slide technology/whole slide images (WSI) [2, 20, 35], and one used both real-time and virtual slide technologies [37]. In one study [23] the slides were transmitted to a password-protected account where the cytopathologists could view them, one used a hospital website [39], two of them used NetCame software [32, 33], one used Caseviewer [20], one used Skype [14], one used WhatsApp [36], one used a Cloud-based portal software [35], three used a Web Browser [13, 34, 38], one used an NDP server [2], and one did not define the modality of transmission [37].

Twelve studies examined the diagnostic concordance of TC diagnosis with final cyto-pathological diagnosis [2, 13, 14, 20, 23, 32–38], seven analyzed TC application on ROSE for preliminary adequacy assessment of the samples [14, 20, 32–34, 37, 39], four evaluated TC image quality [13, 14, 35, 38], two examined the use of TC in preliminary diagnosis [20, 32] and two evaluated the use of TC in acquiring a second opinion [13, 35]. Four studies reported the diagnostic time when TC was used: Khurana et al. [33] estimated 4 to 6 min for each slide pass using real-time microscopy; Yao et al. [37] reported that the average time spent per slide was 270 s (about 4.5 min) using VisionTek Digital Microscope (VDM) real-time microscopy and 122 s (about 2 min) for single Z stack digital scan (SZDS) virtual microscopy, compared to 113 s (about 2 min) for conventional light cytology; Sahin et al. [36] reported an average time of 4.25 min for image capture of thyroid FNAB and 2.14 min from image transfer to diagnosis using static images; Mosquera-Zamudio et al. [35] reported that the average time spent to read and interpret a case (including relevant metadata of the patient) was 6.2 min using virtual microscopy.

A comprehensive view of the characteristics of the included studies is provided in Table 3S in the Online resource. Table 1 summarizes the main results of this review.

**Table 1:** Summary of results

Outcome	Main results	GRADE quality of evidence
Diagnostic concordance between diagnosis via telecytology and conventional cytology	The concordance rate between TC and conventional cytology was excellent in 7/11 (63.6%) studies. Most disagreements were found among the indeterminate and follicular neoplasm cases. Low-cost methods showed good concordance	Moderate
Preliminary assessment of adequacy of samples	TC can be used to perform preliminary assessment and the non-diagnostic rate drops when adequacy of samples is evaluated with TC. The concordance rate of the adequacy assessment of samples between TC and conventional cytology was generally high, ranging from 74 to 100%	Moderate
Evaluation of telecytology image quality	Image quality was referred to as perfect or nearly perfect in most cases, regardless of telecytology technique	High

### Diagnostic concordance between diagnosis via telecytology and conventional cytology

Twelve studies reported data on concordance rates [2, 13, 14, 20, 23, 32–38]: four studies used static images [13, 23, 36, 38], four used whole slide images (WSI) [2, 20, 35, 37], and five used real-time TC [14, 32–34, 37]. Nine of them compared TC with conventional cytology [2, 14, 23, 32–37], one with histology [20] and two had data on concordance with both conventional cytology and histology [13, 38].

To evaluate the magnitude of the concordance rate, the values of kappa statistics were interpreted as follows: poor agreement (k between 0.00 and 0.20), fair agreement (k between 0.20 and 0.40), moderate agreement (k between 0.40 and 0.60), good agreement (k between 0.60 and 0.80), and excellent agreement (k between 0.80 and 1) [35, 40]. Three studies reported results only with the percentage of agreement, without calculating the k statistic, but all values were above 90% of agreement and were considered excellent.

The concordance rate between TC and conventional cytology was excellent in 7/11 (63.6%) studies [13, 23, 32–34, 36, 38], good in one (9.1%) study [14], and moderate in one (9.1%) [2], with only one (9.1%) study reporting a poor concordance rate [35]. The study from Yao et al. [37] reported two different intraobserver agreements from the two cytopathologists involved in the study (with the intraobserver agreement being the concordance rate between two different techniques performed by the same pathologist): the cytopathologist expert in TC had an excellent concordance rate with a k between 0.85 and 0.93 depending on the technique used (real-time TC and WSI respectively), whereas the other cytopathologist, with less exposure to digital pathology, had a good concordance rate of 0.7 and 0.75.

Some studies also analyzed the diagnostic categories independently, with the categories being benign, indeterminate, follicular neoplasm, suspicious for malignant, and malignant [2, 13, 14, 32, 34]. Most disagreements were found among the indeterminate and follicular neoplasm cases, whereas the other three categories always resulted in a good or excellent concordance rate.

If we divide the studies according to the TC technique used, static image studies [13, 23, 36, 38] always had an excellent concordance rate. Three (60%) real-time TC studies [32–34] reported an excellent concordance rate and one (20%) [14] found a good concordance rate; additionally, Yao et al. [37] showed excellent or good agreement when the expert or the less skilled cytopathologist was involved, respectively. Among the studies that used WSI, the data varied: Yao et al. [37] found an excellent or good concordance rate depending on exposure to TC (k = 0.93 for the expert and k = 0.75 for the other); Gerhard et al. [2] found a moderate concordance rate, while Mosquera et al. [35] found a poor agreement.

The interobserver agreement, meaning the concordance rate between different cytopathologists on the same TC diagnosis, was reported in 3 studies with different results. Georgoulakis et al. [23] used static images and found an excellent interobserver agreement. Additionally, when the analysis was conducted in serial rounds, where the cytopathologists analyzed the same slides multiple times, the interobserver agreement, even if always excellent, improved slightly over time from a mean k = 0.89 in the first round to a mean k = 0.91 in the third one. Yao et al. [37] and Gerhard et al. [2] used the WSI technique and reported a good (k = 0.70) and a moderate interobserver agreement (k = 0.57) respectively; the same study from Yao et al. [37] also used real-time TC and found a moderate interobserver agreement (k = 0.47).

Three studies [13, 20, 38] compared TC diagnosis with the final histological diagnosis and always showed good reproducibility between the two techniques. More importantly, the accuracy rates with TC (sensitivity, specificity, positive predictive value, negative predictive value, and total accuracy) were comparable to those found with conventional cytology: Archondakis et al. [38] measured accuracy of diagnosis based on static images and conventional cytology compared with post-thyroidectomy histological diagnosis, and found that the two accuracies were similar (with a total accuracy of 97.21% and 97.11% respectively); Canberk et al. [20] compared their results with the one obtained in large series using conventional cytology (Bongiovanni et al. [41] and Lee et al. [42]) and found very similar results (total accuracy of 94% versus 69% and 95% respectively).

Additionally, the study from Canberk et al. [20], which used TC for primary diagnoses of thyroid FNAB, was able to compare its results with the benchmarks of the Bethesda System and found a distribution of cases (benign, indeterminant, follicular neoplasm, suspicious malignant, and malignant) and a total surgical rate in line with the suggested annual benchmarks.

Two studies [14, 36] evaluated low-cost methods of TC, meaning they only used a smartphone to capture the images and transmitted them with free software (one used “WhatsApp”[36] and the other “Skype” [14]). Sahin et al. [36] used static images as TC technique, while Costa et al. [14] used a real-time ROSE assessment. In both cases, they found a good concordance between TC diagnosis and conventional cytology, with a respective intraobserver agreement of 78.85% (k = 0.839) and 83.3% (k = 0.685).

### Preliminary assessment of adequacy of samples

Seven studies reported data on the use of TC for preliminary assessment of adequacy of thyroid FNAB samples [14, 20, 32–34, 37, 39]. They generally showed that TC can be used to perform preliminary assessment and that the non-diagnostic rate drops when adequacy of samples is evaluated with TC. Five studies [14, 20, 33, 34, 37] showed that the concordance rate of the adequacy assessment of samples between TC and conventional cytology was generally high, ranging from 74 to 100%.

Using WSI, TC Canberk et al. [20] reported an interobserver agreement rate between TC (performed by a cytotechnologist) and conventional cytology (performed by a pathologist) of 88% (24 out of 25 cases). When real-time microscopy was used, Khurana et al. [33] reported that the number of cases that were considered unsatisfactory by TC was 21 out of 100: 4 out of the 21 nodules were considered benign on final cytological assessment and the remaining 17 cases were categorized as unsatisfactory also on conventional cytology. Trabzonlu et al. [34] made a two-phase study. During the “test” phase, cytopathologists were made comfortable with the process and could view the slides in the same room by a TV screen without communicating with one another, performing only the adequacy assessment. During the second phase, diagnostic categories and specific diagnoses were required. They reported a concordance rate of 83.3% in the first phase of the study and 94.8% in the second one.

Lin et al. [39] showed that the unsatisfactory rate dropped significantly (from 8.8% to 3.8%) when telecytology ROSE was introduced in a center that didn't perform preliminary assessment of FNAB. Additionally, there was no difference in the Unsatisfactory/Non-Diagnostic rates between TC ROSE and conventional ROSE. Izquierdo et al. [32] also reported a drop in the unsatisfactory rate in the group in which samples were transmitted with TC for adequacy assessment compared to those which weren’t, but it didn’t reach statistical significance.

Yao et al. [37] evaluated how the intraobserver concordance rate varied with real-time VDM, or virtual slide SZDS compared to conventional cytology. The comparison was made between two different cytopathologists; the intraobserver agreement rate was 0.94 and 0.74 between conventional cytology and VDM, whereas it was 1 and 0.86 between conventional cytology and SZDS respectively for cytopathologists A and B.

### Evaluation of telecytology image quality

Four studies analyzed image quality, using static images [13, 38], WSI [35], and real-time images [14]. In all cases, the quality was subjectively evaluated by the cytopathologists who performed the diagnosis. Image quality was referred to as perfect or nearly perfect in most cases, regardless of telecytology technique. The three studies with digital images evaluated image quality on a scale of 1–10; in the two studies with static images, all the participants reported a really high image quality, with a mean score of 9.5 [13] and 9.1 [38] respectively, while Mosquera et al. [35] reported an average image quality score of 8.3 for WSI without Z-stack and 8.7 if Z-stack was used.

Costa et al. [14] described the image quality of low-cost real-time telecytology. They analyzed the delay in image transmission, clarity of the image, and clarity of voice command, classifying the image as “excellent” when all three parameters worked flawlessly, “good” when only one parameter presented issues, and “poor” when there were problems with two or more parameters. Given that, the study reported an excellent quality of transmission in 57% of cases and a good quality in 24% of cases, with only 19% of cases reported as poor. Additionally, they examined differences in the concordance rates depending on the quality of transmission: poor, good, and excellent quality of transmission respectively had a concordance rate of 62.5% (k = 0.500), 77.8% (k = 0.625), and 88% (k = 0.774).

## Discussion

The majority of the studies we analysed consistently demonstrated a high concordance rate between telecytology and conventional cytology, both in the preliminary assessment of adequacy of samples and in diagnostic concordance. The majority of studies reported excellent or good agreement rates and these findings suggest that telecytology could be a robust and reliable tool for pathology departments.

Interestingly, studies employing WSI as the telecytology technique demonstrated comparable or lower concordance rates compared to studies using static images or real-time TC. This result may be counterintuitive, as WSI allows for a more comprehensive analysis of the entire slide. On the other hand, with real-time TC and even more so with static images, the on-site cytotechnologist/cytopathologist is responsible for selecting the best areas for the off-site pathologist to view. In the analysed studies, the on-site personnel were already expert in thyroid FNAB and familiar with the telecytology technique, ensuring a good selection of fields. Whereas Archondakis et al. and Georgoulakis et al. [23, 38], using static images, defended their choice and the importance of an expert cytopathologist to achieve a reliable result, Gerhard et al. [2], using WSI, suggested a potential selection bias in the other studies, where selecting representative areas may facilitate the diagnosis. In real-time TC this problem is reduced, as the on-site personnel are often guided by pathologist off-site, minimizing image selection.

It should be noted that studies reporting lower agreement rates with WSI involved pathologists with various levels of experience in both thyroid FNAB and telecytology, while other studies often utilized experienced pathologists for both slide scanning and analysis. This may be relevant, as revealed in the study from Yao et al., where the pathologist with more experience in TC demonstrated a significantly higher intraobserver concordance rate between conventional cytology and both real-time and WSI TC, compared to the other cytopathologist with less exposure to digital pathology [37]. Accordingly, the studies that had an initial validation phase, where pathologists were able to familiarize themselves with the technology, or whose pathologists were already familiar with it, had better concordance rates in the subsequent analysis [20, 34]. We can say that effective TC usage, particularly WSI technology, needs practice in order to be efficient, and gives better results when used by expert pathologists. On the other hand, in the case of static images, the off-site pathologists may be less competent in the context of TC, but it is very important that the cytotechnologist/cytopathologist on-site chooses the best representative images to ensure a correct analysis off-site.

Regardless of the technique used, most of the discrepancies between the TC diagnosis and that of conventional cytology, when referring to the Bethesda system, were observed in the atypia of undetermined significance/follicular lesion of undetermined significance (AUS/FLUS) and in the suspicious for follicular neoplasia/follicular neoplasia groups (SFN/FN), whereas benign, suspicious for malignant, and malignant groups always had an excellent concordance rate [2, 14]. This is expected, since even with conventional cytology the former categories are the ones that cause the most discrepancies between cytopathologists.

Interestingly, Archondakis et al. with static images and Canberk et al. with WSI made a comparison between TC diagnosis and the final histological diagnosis and compared those results with conventional cytology. In both cases TC diagnostic accuracy was comparable to that of conventional cytology [20, 38].

TC also showed some potential in preliminary diagnosis. Most of the benign and malignant lesions were well recognized and described. Discrepancies among the pathologists were mostly in undetermined or uncertain cases and some of them, when analyzed with conventional cytology after the TC, were downgraded from malignant to benign, whereas there was never an upgrade from benign to malignant [20, 32].

Regarding the preliminary assessment of samples, all the studies showed a high concordance rate, indicating that using TC-ROSE can be a successful alternative to conventional ROSE. The introduction of TC-ROSE has proven to reduce the unsatisfactory rate in centres without a pathologist available for immediate assessment of FNAB samples [32, 39], and the magnitude of this reduction is comparable to that achieved by standard ROSE [39]. This finding may have significant implication for small and remote facilities that lack on-site pathologists, as they need to send their samples directly to other larger facilities for cytological analysis. By introducing TC in these centers, with the assistance of just a cytotechnologist to prepare the samples and manipulate the microscope while the pathologist remotely views the images, adequacy can be immediately assessed, and the number of passes can be reduced [43]. Furthermore, the reduction in unsatisfactory rates leads to fewer patients requiring re-biopsy, thereby reducing waiting times for an accurate diagnosis, and lowering the cost of multiple procedures [39]. Since the pathologist, even in a remote setting, can make legally valid preliminary diagnoses, patient anxiety can be reduced if the initial assessment suggests a benign condition [32, 33]. Conversely, patients can be promptly referred to specialized centers if malignancy is suspected, expediting the start of appropriate therapy.

The included studies revealed that the TC image quality is consistently high, regardless of the technique used, for both static and dynamic technologies.

### Implications and possible practical applications

Our study suggests that TC could be a suitable option for the daily diagnostic workflow of thyroid nodules FNAB. Given that accuracy of this technique is highly dependent on the skills and experience of the involved personnel, adequate training on the technical aspects of TC systems used is crucial and it should be tailored to the professional figure involved (pathologists [44], residents [45], cytotechnologist [46]).

Static TC systems may be preferred because of the higher costs, telecommunication, and transmission issues associated with the use of dynamic and virtual-slide systems [38, 47]. TC has also proven to be effective with low-cost methods; two studies used just a smartphone to scan images from the microscope and showed a good agreement rate with both static and real time images, using simple and free methods for transmission of data such as WhatsApp and Skype [14, 36]. This suggests that TC could be useful also in remote locations with limited resources, lacking a pathologist, or in need of a second opinion on a challenging case [48]. Nevertheless, low-cost options should always aim to the best quality achievable, given that quality of transmission also influences concordance rate [14].

### Strengths and limitations

The main limitation of this review was due to heterogeneity in the included studies: study design, telecytology techniques, data reporting style, and outcomes varied from a study to another; we also did not find a statistical methodological justification nor a previous literature record of an empirical study that would provide us a rationale to perform a quantitative synthesis through meta-analysis.

Additionally, the comparison between telecytology and conventional cytology was sometimes made as an intraobserver agreement, meaning that the same pathologist first made the TC diagnosis and then the conventional cytology diagnosis, but other times the two analyses were made by two different pathologists, as an interobserver agreement. This opens the possibility that the two cytopathologists didn’t agree with each other, and the probability of that is not negligible, since cytology is a very subjective analysis. This is proved, for example, by the studies of Yao, in which the two cytopathologists had an agreement rate with conventional glass cytology of 0.67. This value represents a good agreement but is surely not perfect, so it should be expected that the interobserver agreement rate would not be perfect even with TC [37]. We think, in order to have solid and reliable results, analysis should measure the intraobserver agreement between TC and conventional cytology, and then the interobserver agreement between different pathologists using the same TC methods.

Our study represents, to our knowledge, the first record of a systematic review in the field of telecytology, and specifically about thyroid FNAB. Additionally, it provides the most up-to-date evidence on the topic, given that most of the included studies were published during the last 5 years.

## Conclusions

Our review suggests that telecytology could be a valuable implementation for thyroid FNAB evaluation, as the concordance rate with conventional cytology is consistently high, both for primary diagnosis and preliminary assessment of samples. The image quality is consistently high regardless of the technique used and, therefore, it does not represent a limitation for diagnosis.

## Supplementary Information

Below is the link to the electronic supplementary material.

Supplementary file1 (PDF 214 KB)

## Data Availability

Data sharing not applicable to this article as no datasets were generated or analyzed during the current study.
