# Supplementary material for: The use of telecytology for the evaluation of thyroid nodules fine-needle aspiration biopsy specimens: a systematic review
Source: J Endocrinol Invest. 2024 May 4;47(10):2397–406. doi: 10.1007/s40618-024-02378-3 (PMC11393276; doi:10.1007/s40618-024-02378-3)
Supplement: Supplementary file 1 — Supplementary file1 (PDF 214 KB) [file 40618_2024_2378_MOESM1_ESM.pdf]

# The use of telecytology for the evaluation of thyroid nodules fine-needle aspiration biopsy specimens: a systematic review

**Journal of Endocrinological Investigation**

**Vittorio Oteri MD[1*], Simone Piane MD[2], Elisa Cocci MD[3]**

[1]Endocrine Unit, Department of Clinical and Experimental Medicine, University of Catania, Garibaldi-Nesima Hospital, Catania, Italy

[2]Department of Medicine and Surgery, University of Milano-Bicocca, Milan, Italy

[3]Department of Clinical and Experimental Medicine, Marche Polytechnic University, Ancona, Italy

**Correspondence should be addressed:*

Vittorio Oteri, Endocrine Unit, Department of Clinical and Experimental Medicine, University of Catania, Garibaldi-Nesima Hospital, Catania, Italy; email: research@droteri.it

***Appendix 1.*** *Search strategy*

PubMed search strategy:

(((telemedicine OR "Telemedicine"[Mesh] OR Telehealth OR Teleconsultation OR eHealth OR (digital health)) AND (cytology OR cytopathology OR "Cytology"[Mesh])) OR telecytology OR telecytopathology OR Telepathology OR "Telepathology"[Mesh] OR (virtual microscopy) OR (virtual cytology) OR cytotechnology OR (digital image)) AND (Thyroid OR "Thyroid Gland"[Mesh]) AND ((Fine-Needle Aspiration) OR (Biopsy, Fine-Needle) OR (Aspiration Biopsy) OR "Biopsy, Fine-Needle"[Mesh] OR FNAB OR FNA)

Embase and Cochrane Library search strategy:

(((telemedicine OR Telehealth OR Teleconsultation OR eHealth OR (digital health)) AND (cytology OR cytopathology)) OR telecytology OR telecytopathology OR Telepathology OR (virtual microscopy) OR (virtual cytology) OR cytotechnology OR (digital image)) AND (Thyroid) AND ((Fine-Needle Aspiration) OR (Biopsy, Fine-Needle) OR (Aspiration Biopsy) OR FNAB OR FNA)

***Table 1S.*** *Risk of bias assessment of the included non-randomized studies following the ROBINS-I tool*

| Author et al. (Year) | Confounding | Selection of participants into the study | Classification of interventions | Deviations from intended interventions | Missing data | Measurement of the outcome | Selection of the reported result | Overall |
|---|---|---|---|---|---|---|---|---|
| Archondakis et al (2009) | Low | Low | Low | Moderate | Low | Low | Low | **Moderate** |
| Archondakis et al (2021) | Low | Low | Low | Moderate | Low | Low | Low | **Moderate** |
| Canberk at al (2019) | Low | Low | Low | Low | Low | Low | Low | **Low** |
| Costa et al (2018) | Moderate | Low | Low | Low | Low | Low | Low | **Moderate** |
| Georgoulakis et al. (2010) | Low | Low | Low | Low | Low | Low | Low | **Low** |
| Gerhard et al (2013) | Moderate | Low | Low | Moderate | Moderate | Moderate | Low | **Moderate** |
| Izquierdo et al. (2013) | Low | Low | Low | Moderate | Moderate | Low | Low | **Moderate** |
| Khurana et al (2011) | Low | Low | Low | Moderate | Low | Low | Moderate | **Moderate** |
| Lin et al. (2020) | Low | Low | Low | Low | Low | Low | Low | **Low** |
| Mosquera-Zamudio et al. (2019) | Moderate | Moderate | Low | Low | Moderate | Moderate | Moderate | **Moderate** |
| Sahin et al (2018) | Moderate | Moderate | Low | Low | Low | Low | Low | **Moderate** |
| Trabzonlu et al. (2022) | Moderate | Low | Moderate | Low | Serious | Low | Moderate | **Serious** |
| Yao et al. (2018) | Moderate | Low | Low | Moderate | Moderate | Low | Low | **Moderate** |

***Table 2S.*** *Quality assessment of the outcomes following GRADE approach*

| Outcome | Study design | Risk of Bias | Inconsistency of results | Indirectness of evidence | Imprecision | Publication bias | Large magnitude of effect | Dose-response gradient | Plausible confounding | Quality |
|---|---|---|---|---|---|---|---|---|---|---|
| Diagnostic concordance between diagnosis via telecytology and conventional cytology | Observational | Unclear | Undetected | Undetected | Undetected | Undetected | N/A | N/A | No | **Moderate** |
| Preliminary assessment of adequacy of samples | Observational | Unclear | Undetected | Undetected | Undetected | Undetected | N/A | N/A | No | **Moderate** |
| Evaluation of telecytology image quality | Observational | Low | Undetected | Undetected | Undetected | Undetected | N/A | N/A | No | **High** |

N/A – Not applicable;

***Table 3S.*** *Characteristics of the included studies*

| Author et al. (Year) | Country | Type of study | Number of thyroid cases | Telecytology technique | Main Findings |
|---|---|---|---|---|---|
| Archondakis et al. (2009) | Greece | Retrospective | 252 | Static images | Concordance rate between TC and CC had a k=0.94. Diagnostic accuracy of TC and CC compared with Histology diagnosis was similar (always above 96.69%). Image quality was really good in more than 80% of cases. |
| Archondakis et al. (2021) | Greece | Retrospective | 141 | Static images | Concordance rate between the 3 cytopathologist for benign and malignant lesions had k=0.85, k=0.84 and k=0.88, and for indeterminate lesions k=0.73 and k=0.78. Interobserver reproducibility between TC and Histology had a k=0.75. Image quality was nearly perfect or perfect for most of the images. |
| Canberk et al. (2020) | Turkey | -validation phase Retrospective -study phase Prospective | validation phase: 25 study phase: 227 | Virtual slide WSI | Adequacy agreement between TC and CC was 88% in the validation phase. Diagnostic accuracy compared with Histology ranged from 80% to 100%, dependending on diagnostic cathegories in the study phase. |
| Costa et al. (2018) | Brazil | Prospective | 42 | Real-time mycroscopy with a smartphone | Adequacy agreement between TC and CC was 90.5% (k=0.461). Diagnostic agreement between TC and CC was 83.3% (k=0.685). Transmission quality was excellent or good in the majority of cases, poor in 19%. The quality influenced agreement rate: Low quality k=0.5, good k=0.625, excellent k=0.774. |
| Georgoulakis et al. (2011) | Greece | Retrospective | 270 | Static images | Interobserver agreement between TC and the initial CC was very high in all the rounds (k =0,921-0,955). Interobserver agreement in the initial rounds and in the reviews was nearly perfect and improved over the course of the review rounds (k = 0,870-0,939). Intraobserver agreement (between the checking and the review rounds) ranged within k =0,967-1 depending on the pathologist's experience. |
| Gerhard et al. (2013) | Portugal | Retrospective | 222 | Virtual slide WSI | Intraobserver agreement between TC and CC was 77.5%, with k=0.54, with more non-diagnostic cases in TC (20.3% vs 8.1%); concordance rate for malignant and benign lesions was high (75% and 83.3%). Interobserver concordance rate was 80.2% with k=0.57, it was 0.0% for indeterminate lesions, and 93.3% and 100% respectively for benign and malignant lesions |
| Izquierdo et al. (2013) | USA | Retrospective | 92 | Real-time mycroscopy | Concordance rate between TC preliminary diagnosis and the CC was excellent for benign and malignant cases in the transmitted group, the only discrepancies were in the unsatisfactory group. |
| Khurana et al. (2011) | USA | Retrospective | 100 | Real-time mycroscopy | Accuracy rate between TC and CC was 94%. Unsatisfactory rate with TC was 21%, reduced to 17% in final cytologic evaluation. |
| Lin et al. (2020) | USA | Retrospective | 2387 | Real-time mycroscopy | Unsatisfactory rate in the study group after the introduction of ROSE and TC ROSE lowered significantly. In the control group it remained unchanged. Adequacy assessment agreement between remote ROSE and final adequacy assessments was 97% (242/250) and k= 0.699. |
| Mosquera-Zamudio et al. (2019) | Colombia | Retrospective | 10 (65% of total cases) | Virtual slide WSI | Concordance rate between the TC and CC ranged between 36 and 71%, Interobserver agreement was 93% for malignant cases, 79% for benign, and 64% for indeterminate. Overall interobserver agreement was poor (k= 0.152, P< 0.0001). Image quality scores were 8.3 for Hamamatsu WSI and 8.7 for Panoptiq slides. |
| Sahin et al. (2018) | Turkey | Retrospective | 52 (30% of total cases) | Static-images with a smarthphone | Concordance rate between TC and CC was 78.85% (k=0.839). Patient management changed with TC diagnosis in 15.38% of cases |
| Trabzonlu et al. (2022) | USA | Retrospective | 20 (38% of total cases) | Real-time microscopy | Adequacy concordance rate in the first case set was 83.3%. In the second case set the adequacy concordance rate was 94.8%, the diagnostic category concordance was 91.9%, the specific diagnosis concordance was 88.1%. |
| Yao et al. (2018) | USA | Retrospective | 16 (27% of total cases) | Real-time microscopy; Virtual slide SZDS | For adequacy assessment the interobserver agreement was higher for CLM and SZDS (k = 0.74). The intermodality agreement was k=0.94 and 1 for cytopathologist A (CLM vs VDM and CLM vs SZDS) and 0.74 and 0.86 for Cytopathologist B. For preliminary diagnosis the interobserver agreement was higher for SZDS (k=0.70). The intermodality agreement was k=0.85 and 0.93 for Cytopathologist A and 0.7 and 0.75 for Cytopathologist B. |

CC - Conventional Cytology; CLM - Conventional Light Microscopy; ROSE - Rapid On Site Evaluation; SZDS - Single Z-stack Digital Scan; TC – Telecytology; VDM - VisionTek Digital Microscope; WSI - Whole Slide Imaging.
